# Application of MR Imaging Features in Differentiation of Renal Changes in Patients With Stage III Type 2 Diabetic Nephropathy and Normal Subjects

**DOI:** 10.3389/fendo.2022.846407

**Published:** 2022-05-04

**Authors:** Baoting Yu, Chencui Huang, Xiaofei Fan, Feng Li, Jianzhong Zhang, Zihan Song, Nan Zhi, Jun Ding

**Affiliations:** ^1^ Department of Radiology, China–Japan Union Hospital of Jilin University, Changchun, China; ^2^ Department of Research Collaboration, R&D Center, Beijing Deepwise & League of PHD Technology Co., Ltd., Beijing, China; ^3^ Department of Radiology, Chang Chun Central Hospital, Changchun, China

**Keywords:** radiomics, texture analysis, diabetic nephropathy, magnetic resonance, renal changes

## Abstract

**Objective:**

The objective of the study was to explore the value of MRI texture features based on T1WI, T2-FS and diffusion-weighted imaging (DWI) in differentiation of renal changes in patients with stage III type 2 diabetic nephropathy (DN) and normal subjects.

**Materials and Methods:**

A retrospective analysis was performed to analyze 44 healthy volunteers (group A) and 40 patients with stage III type 2 diabetic nephropathy (group B) with microalbuminuria. Urinary albumin to creatinine ratio (ACR) <30 mg/g, estimated glomerular filtration rate (eGFR) in the range of 60–120 ml/(min 1.73 m^2^), and randomly divided into primary cohort and test cohort. Conventional MRI and DWI of kidney were performed using 1.5 T magnetic resonance imaging (MRI). The outline of the renal parenchyma was manually labeled in fat-suppressed T2-weighted imaging (FS-T2WI), and PyRadiomics was used to extract radiomics features. The radiomics features were then selected by the least absolute shrinkage and selection operator (LASSO) method.

**Results:**

There was a significant difference in sex and body mass index (BMI) (P <0.05) in the primary cohort, with no significant difference in age. In the final results, the wavelet and Laplacian–Gaussian filtering are used to extract 1,892 image features from the original T1WI image, and the LASSO algorithm is used for selection. One first-order feature and six texture features are selected through 10 cross-validations. In the mass, 1,638 imaging extracts features from the original T2WI image.1 first-order feature and 5 texture features were selected. A total of 1,241 imaging features were extracted from the original ADC images, and 5 texture features were selected. Using LASSO-Logistic regression analysis, 10 features were selected for modeling, and a combined diagnosis model of diabetic nephropathy based on texture features was established. The average unit cost in the logistic regression model was 0.98, the 95% confidence interval for the predictive efficacy was 0.9486–1.0, specificity 0.97 and precision 0.93, particularly. ROC curves also revealed that the model could distinguish with high sensitivity of at least 92%.

**Conclusion:**

In consequence, the texture features based on MR have broad application prospects in the early detection of DN as a relatively simple and noninvasive tool without contrast media administration.

## 1 Introduction

Diabetes mellitus (DM) is the third leading cause of death in China after cardiovascular disease and malignant tumor. Diabetes is one of the fastest growing diseases worldwide, projected to affect 693 million adults by 2045 (1).One of the most important chronic complications of diabetes is diabetic nephropathy (DN). Diabetic nephropathy (DN) is a major cause of end-stage renal disease throughout the world in both developed and developing countries (2). Studies have shown that early diagnosis of DN can reduce the risk of progression to end-stage renal disease by 80% (3).Therefore, early diagnosis and treatment is of great significance to improve the survival rate and prognosis of DN patients.

Diabetic nephropathy (DN) has been surging as the leading cause of end-stage renal disease (ESRD), as approximately one-half, in Europe, United States, Japan, and Taiwan (4). In clinical practice, we found that due to the strong compensatory ability of the kidney itself, some DN patients actually have more serious kidney damage even in mild abnormal GFR or microalbuminuria, but only invasive biopsy can be found at this time. So looking for a noninvasive and accurate way to reflect DN staging has become the direction to improve the prognosis of DN patients. Although the imaging examination of the kidney has been a big step forward for the noninvasive evaluation of the kidney, most of them still stay at the morphological level. The role of magnetic resonance imaging (MRI) in renal magnetic resonance imaging sequence in the early diagnosis of diabetic nephropathy is limited. Functional MRI does not significantly prolong the examination time, or require preparation of patients, as these are non-invasive imaging modalities capable of providing quantitative parameters to assess the change of renal microstructure and function (5). Therefore, it will be helpful to find a safe and repeatable method to reflect the early renal changes.

Functional MRI scan techniques, diffusion tensor imaging (DTI), and diffusion-weighted imaging (DWI) may provide early noninvasive detection of functional changes in the kidney by following the diffusion of water molecules (6). A multitude of previous studies have proven the correlation of DTI and DWI quantitative parameters (fractional anisotropy (FA) and apparent diffusion coefficient (ADC)) with estimated glomerular filtration rate (eGFR), glomerulosclerosis, tubular damage, and interstitial fibrosis (7–10) due to which these parameters could be useful for the evaluation of diabetic patients with early DN (11).

Through the postprocessing of medical images with computers, compared with human eyes, imaging texture features can express a lot of information to us. After that, we can establish a radiomic model for diagnosis and prediction based on this. This model is based on texture features, which can be used to diagnose diseases and predict therapeutic effects (12–16). The goal of this study was to analyze the value of combining routine MR and DWI texture analysis in identifying the renal changes in patients with stage III type 2 DN and normal subjects. In our research, we analyzed the texture features of normal kidney MRI images and stage 3 DN. We found that early renal damage in diabetic patients can be detected by radiomics because of its good effect.

## 2 Materials and Methods

### 2.1 Patients

A total of 40 patients (12 women, 28 men) with stage III diabetic nephropathy and 44 healthy controls (31 women, 13 men) were collected in the China–Japan Union Hospital of Jilin University from Jan. 2020 to Sep. 2021, and we randomly divided them into a primary cohort (56 patients) and a test cohort (28 patients). The preoperative MRI examination of DN confirmed by biopsy was obtained from January 2020 to 2021. The clinical characteristics of our study cohort are shown in **Table 1**.

**Table 1:** Patient characteristics in the primary and test cohort (n = 72).

	Primary cohort				Test cohort			
	DN (n = 27)	NP (n = 29)	Statistic value	*P*-value	DN (n = 13)	NP (n = 15)	Statisticvalue	*P*-value
**Sex n (f)**								
Male	20 (71.43%)	9 (31.03%)	*χ^2^* = 9.301	0.002*	8	4	*χ^2^* = 3.458	0.063
Female	7 (28.57%)	20 (68.97%)			5	11		
**Age, years**								
Range	32–78	32–69	F = 3.231	0.078	38–72	45–62	F = 6.858	0.99
Mean ± SD	52.79 ± 2.295	47.41 ± 1.927			53.15 ± 3.109	53.20 ± 1.631		
**BMI**								
Normal	8 (28.57%)	20 (68.97%)	*χ^2^* = 9.301	0002*	3	6	*χ^2^* = 0.914	0.339
Fat	20 (71.43%)	9 (31.03%)			10	9		

A P-value < 0.05 was considered to indicate a statistically significant difference. *Sex, BMI's test ^ chi-square test, *age's test ^ independent-samples t-test.

The inclusion criteria were clinically diagnosed as DN stage III with complete clinical data, no concomitant diseases, can cooperate with MRI examination, a history of DM2, stable comorbidities, treatment regimen, oral hypoglycemics, without change in weight, diet, lifestyle and medication therapy for comorbidity in the last 6 months. The main inclusion criteria for the control group were adult patients of both sexes in the age range of 30–70 years, without DM, hypertension, heart or kidney disease. The exclusion criteria for all groups were pregnancy and lactation, liver disease, infectious diseases, malignant diseases, patients with extremity amputations, urinary tract infections, or some other disease, cardiac insufficiency, patients with absolute and relative contraindications for MR imaging (absolute contraindications: patients with pacemakers or with ferromagnetic foreign bodies; relative contraindications: claustrophobia, restless patients). Due to the presence of artifacts, post-processing MRI quality is not ideal.

### 2.2 Nuclear Magnetic Resonance Imaging and Texture Examination

**Figure 1:**
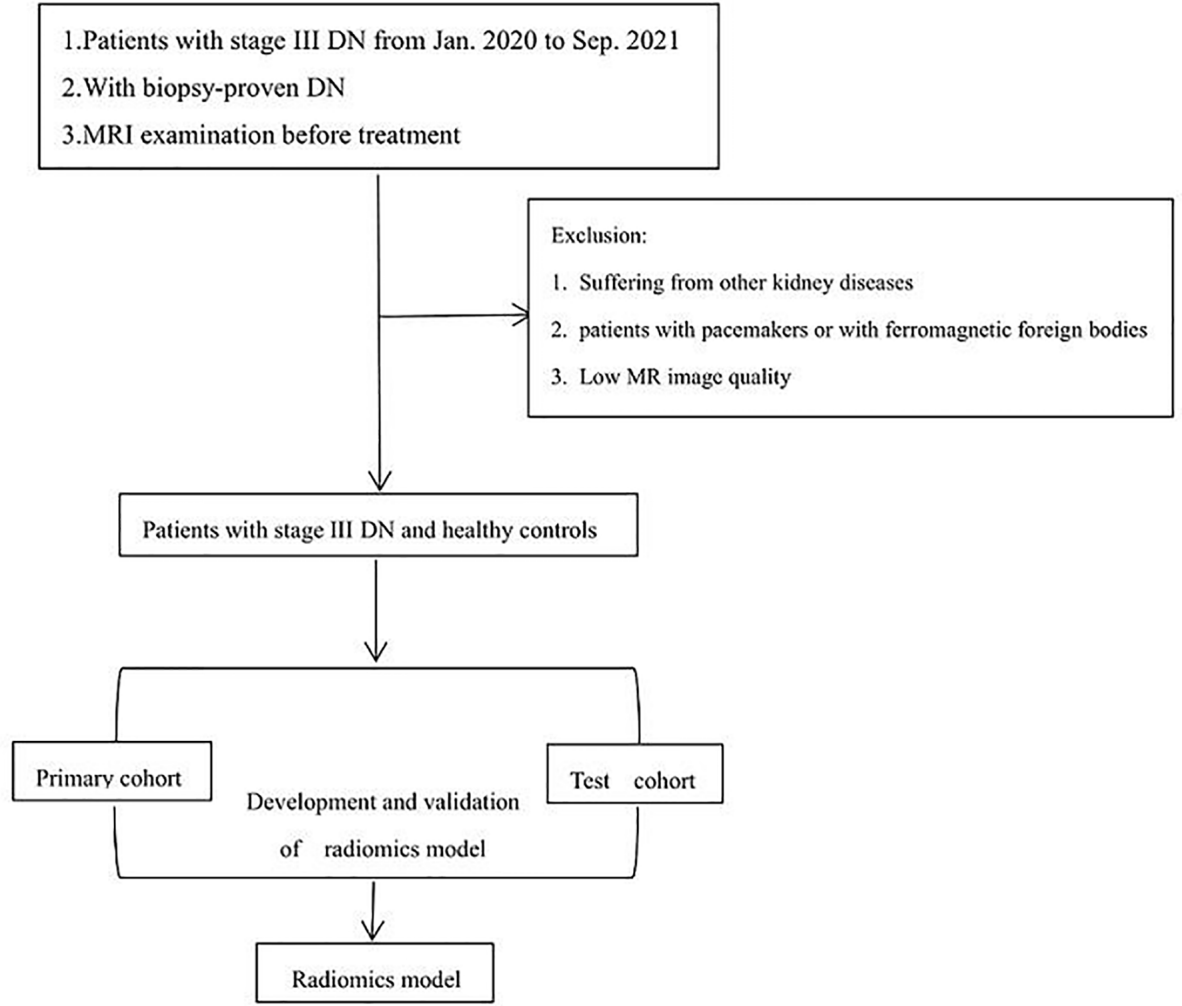
Basic flow chart of this experiment.

MRI was performed by using a 1.5-T Avanto scanner (Siemens Healthineers, Erlangen, Germany) and an 8-channel phased array abdominal coil. In order to ensure good contact under the coil, we use a relaxation pad to fix the patient for non-contrast axial T1WI, FS-T2WI, coronal T2WI, DWI and ADC sequences. The non-contrast axial FS-T2WI sequence is obtained by using the following parameters: repetition/echo time 5,080/87 ms, slice thickness/interlayer gap 4.0/0.4 mm, 20 slices, matrix 256 × 320. In this study, our texture analysis includes image annotations obtained using the Dr. Wise Multimodal Research Platform (https://keyan.deepwise.com). In **Figure 1** we can see the steps of texture analysis.

#### 2.2.1 Delimitation of Tumor ROI

**Figure 2:**
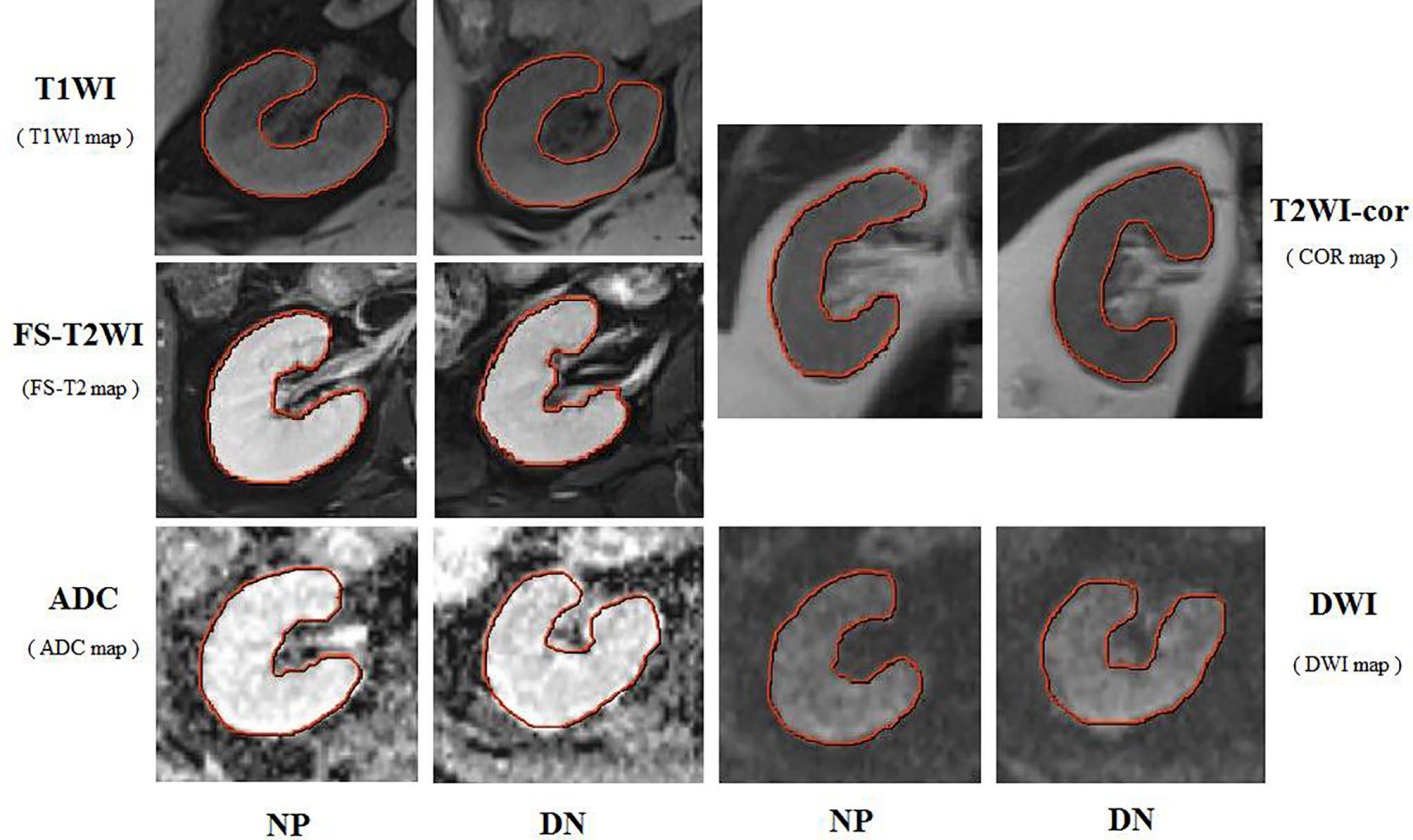
Respective MRI images of the right kidney with stage III type 2 DN and normal persons (NP). The ROI were curved over the renal parenchyma of the right kidney (red curve). In this study, the images analyzed were the T1WI maps, FS-T2WI and T2WI-cor maps, DWI and ADC maps.

We used the original DICOM format for retrospective review, loading and processing of all scans, and then transmitted the results to the post-processing working station. The left kidney is susceptible to heart and respiratory artifacts. To avoid possible image artifacts resulting from air in the digestive tract, the imaging data of the right kidney was analyzed. The ROIs were initially delineated around the outline of the renal parenchyma by two radiologists who had more than 10 years of experience in abdominal MRI interpretation and were blinded to the clinical details. The experts have been trained for three months based on the principle of not exceeding the lesion boundary. Then we get the volume of interest for feature extraction and quantification. The shaft T1WI, FS-T2WI, coronal T2WI, DWI, and ADC scans were selected as the labeling images. The cortex and medulla can be distinguished according to the principle of not exceeding the boundary of the cortex (**Figure 2**).

#### 2.2.2 Extracting Features From MRI Scans

We used high-pass or low-pass wavelet filters and Laplacian-Gaussian filters by different λ parameters to preprocess the original image. Wavelet filtering produces eight decompositions in each stage. In three-dimensional preprocessing, we use all possible combinations of high-pass or low-pass filters (wavelet LLH, wavelet LHL, wavelet LHH, wavelet HLL, wavelet HLH, wavelet HLH, wavelet HHL, wavelet HHHH and wavelet LLL) and four Laplacian-Gaussian filters (λ = 2,3,4,5).These first-order functional components based on these pixel values and the internal and surface texture features were extracted from each ROI of the original images, texture features, namely, the GLCM (gray hierarchical co-occurrence matrix), GLRLM (gray horizontal run matrix), GLSZM (gray level size zone matrix), and GLDM (gray energy level dependent matrix). For the grand total, we extract the radiation features from each ROI, then subtract the average value and divide the standard deviation to obtain the standardized Z score. Then the feature of poor consistency between different groups was removed and the intra-group correlation coefficient (ICC) was calculated. We then select ICC >0.75 features and modeling. Finally, we conduct feature reduction and selection, and use the LASSO (minimum absolute shrinkage and selection operator) algorithm to carry out. This study selected several sequences, namely, non-contrast axial T1WI, FS-T2WI, coronal T2WI, DWI, and ADC. The optimal model is selected for combination by DeLong’s test. The most important characteristics of nonzero coefficients are determined for modeling and improving model performance. The nesting method uses the shrinkage (regularization) process, which penalizes the coefficients of the regression variables and shrinks some of them to zero. A section of the model is selected by variables with non-zero coefficients after contraction. The goal of the process is to minimize the forecasting error.

#### 2.2.3 Establishment of the Model

The texture features were selected to construct the radiomics model to eliminate the redundant features. Logical regression (LR) classifier is used to establish the model through 10-fold cross-validation method. All data are divided into 10 parts, of which 9 are used for model training and the other for evaluating the effectiveness of the model. Finally, all data are used for a primary set and an internal test set. The radiomics labels (i.e., RS-T2WI, RS-ADC) based on T1WI, FS-T2WI, coronal T2WI, DWI, and ADC were constructed, and the scores of corresponding radiomics model labels (radscore) were calculated; Radscore is calculated as radscore = intercept + βi·Xi, where Xi represents the final filtered feature value, and βi represents the corresponding weight coefficient of the final filtered feature.

The clinical factors of DN were statistically analyzed by univariate analysis, and the clinical factors related to the onset of DN (*P <*0.05) were selected. Then these selected clinical factors were analyzed by multivariate LR. For the united model construction and verification process: firstly, DeLong test is performed on axial T1WI, FS-T2WI, coronal T2WI, DWI, and ADC sequences, and the optimal model is selected for combination. To test whether the test set performance of one machine learning model can differ significantly from that of another model, we used the DeLong test. According to the AUC value, models with statistical differences were excluded.

### 2.3 Quantitative Analysis

This study uses Scikit learning package (version 2.2.3) to create classification models. We used Matplotlib (version3.1.0) to draw the ROC curve. For statistical analysis of general data we used SPSS for Windows version 24.0 (IBM Corp., Armonk, NY, USA). Chi-square test is used to detect the difference in classification variables between groups. Independent sample t-test is used to test the inter-group difference of quantitative variables. P <0.05 was considered statistically significant. In this study, two independent sample t-tests were used to test the age of two groups; receiver operating characteristic (ROC) was used to analyze the diagnostic efficacy and area under the curve (AUC) of imaging characteristic parameters for stage III DN. The predictive efficacy included sensitivity (SEN), specificity (SPE), accuracy (ACC), positive predictive value (PPV), positive predictive value (PPV), negative predictive value (NPV), AUC and 95% confidence interval (CI) were used. The AUC values of each prediction model were compared by using DeLong test.

## 3 Results

### 3.1 Patients and Radiological Features

**Table 1** summarizes the characteristics of 84 research participants. There were significant differences between the sex and body mass index (BMI) (*P <*0.05) of the two groups in the primary cohort, and no significant difference in age. There were no differences in the test set of all clinical characteristics. We extracted 1,892 imaging features from the original T1W1 image and selected them by wavelet and Laplace-Gaussian filter and LASSO algorithm.1 first-order feature and 6 texture feature selection by 10 times cross validation method. Using the same methods, a total of 1,638 imaging features were extracted from the original T2WI images, 1 first-order feature and 5 texture features were selected. A total of 1,241 imaging features were extracted from original ADC images, and 5 texture features were selected. The feature lists and coefficient charts are shown in **Table 2**. The five models of axial T1WI, FS-T2WI, coronal T2WI, DWI, and ADC were analyzed by DeLong test. There was no statistical difference between T1WI, FS-T2WI, and ADC models, but there was a difference between T1WI, coronal T2WI, and DWI. Finally, T1WI, FS-T2WI, and ADC models were selected by DeLong test to construct the united model.

**Table 2:** The imaging features based on T1WI, FS-T2WI, ADC, and united model.

No.	Imaging features based on T1WI (Intercept)	coef	relative_to_max
1	gradient_glszm_GrayLevelNonUniformity	1.4128	1
2	exponential_firstorder_Minimum	0.9689	0.6858
3	lbp-3D-m2_glszm_GrayLevelNonUniformityNormalized	−0.8933	−0.6323
4	lbp-3D-m1_glcm_Correlation	−0.8416	−0.5957
5	wavelet-HLH_glrlm_ShortRunLowGrayLevelEmphasis	0.6492	0.4595
6	lbp-3D-m1_glszm_LowGrayLevelZoneEmphasis	−0.6481	−0.4587
7	wavelet-HHH_glszm_GrayLevelNonUniformityNormalized	0.1179	0.0834
**No.**	**Imaging features based on FS-T2WI (Intercept)**	**coef**	**relative_to_max**
1	lbp-3D-m2_glszm_SmallAreaHighGrayLevelEmphasis	−1.5453	−1
2	lbp-3D-m2_gldm_DependenceVariance	−1.0865	−0.7031
3	logarithm_glszm_SizeZoneNonUniformity	0.917	0.5934
4	MeanFullBeforeNormalize	−0.7986	−0.5168
5	original_firstorder_Skewness	0.7385	0.4779
6	wavelet-LL_gldm_DependenceEntropy	0.4581	0.2965
**No.**	**Imaging features based on ADC (Intercept)**	**coef**	**relative_to_max**
1	exponential_glszm_GrayLevelNonUniformity	1.4991	1
2	lbp-2D_glrlm_ShortRunLowGrayLevelEmphasis	1.1323	0.7553
3	wavelet-HL_glcm_ClusterShade	−1.0306	−0.6874
4	lbp-3D-k_glrlm_ShortRunEmphasis	0.8828	0.5889
5	lbp-2D_glrlm_ShortRunHighGrayLevelEmphasis	−0.4154	−0.2771
**No.**	**Imaging features based on united model (Intercept)**	**coef**	**relative_to_max**
1	lbp-3D-m2_glszm_SmallAreaHighGrayLevelEmphasis_T2	−1.1194	−1
2	exponential_glszm_GrayLevelNonUniformity_ADC	0.9311	0.8318
3	logarithm_glszm_SizeZoneNonUniformity_T2	0.8222	0.7345
4	lbp-3D-m1_glszm_LowGrayLevelZoneEmphasis_T1	−0.7809	−0.6976
5	lbp-2D_glrlm_ShortRunLowGrayLevelEmphasis_ADC	0.7481	0.6683
6	wavelet-HL_glcm_ClusterShade_ADC	−0.6952	−0.621
7	exponential_firstorder_Minimum_T1	0.6247	0.5581
8	original_firstorder_Skewness_T2	0.6183	0.5524
9	lbp-3D-m2_gldm_DependenceVariance_T2	−0.5763	−0.5148
10	wavelet-LL_gldm_DependenceEntropy_T2	0.4354	0.3889

**Figure 3:**
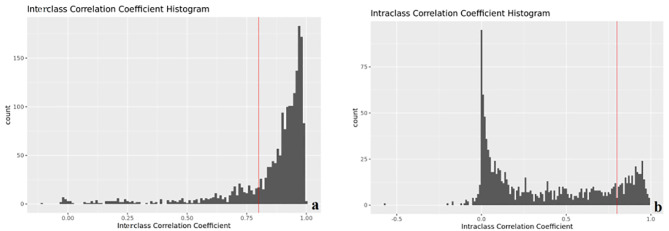
Interobserver **(A)** and intraobserver **(B) **consistency (ICC).

Modeling by LR, the features used to build the model were selected, and the image label based on T1WI, FS-T2WI, and ADC is constructed, two first-order features (original_firstorder_Skewness_T2 and exponential_firstorder_Minimum_T1) and eight texture features (lbp-3D-m1_glszm_LowGrayLevelZoneEmphasis_T1, logarithm_glszm_SizeZoneNonUniformity_T2, wavelet-LL_gldm_DependenceEntropy_T2, lbp-3D-m2_gldm_DependenceVariance_T2, lbp-3D-m2_glszm_SmallAreaHighGrayLevelEmphasis_T2, exponential_glszm_GrayLevelNonUniformity_ADC, lbp-2D_glrlm_ShortRunLowGrayLevelEmphasis_ADC, wavelet-HL_glcm_ClusterShade_ADC) were selected. The interobserver (**Figure 3A**) and intraobserver (**Figure 3B**) of the annotation image has good consistency. All the features of the selected model are consistent >0.8.

### 3.2 Model Assessment

**Figure 4:**
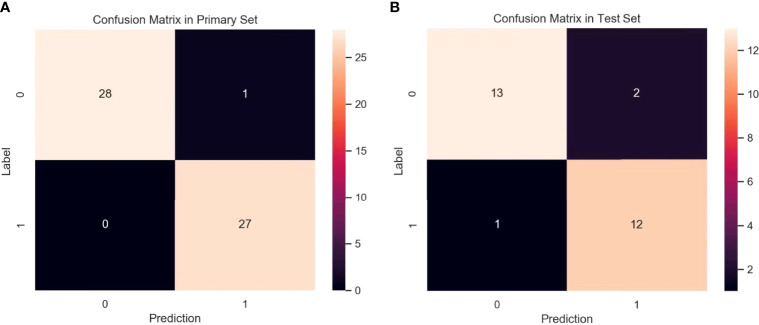
Confusion matrix for the model in primary set **(A)** and test set **(B)**. A confusion matrix was used to examine whether or not there was consistency between the stage III type 2 DN and normal subjects. Different colors represent different cases. The color becomes lighter as the number increases. (Note: Numbers 0 and 1 represent normal patient and DN, respectively).

**Figure 5:**
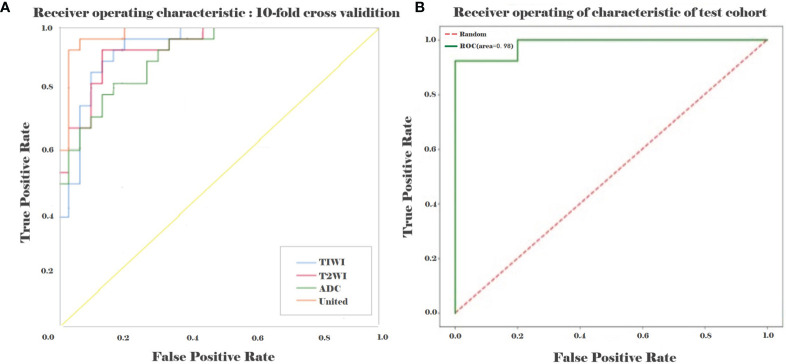
ROC curve analysis of the primary set **(A)** and test set **(B)** between the stage III type 2 DN and normal subjects. The solid lines in different colors indicate that the ROC curve for each model correspond to a different AUC, which represents the positive rate of predict and distinguish the stage III type 2 DN and normal subjects. The solid lines in different colors indicate the ROC curves of the T1WI, FS-T2WI, ADC and united models.

To evaluate the performance of the prediction model in the primary set, firstly, in the internal test, we focus on evaluating the performance of the prediction model. The test data are divided into two types: class distribution equilibrium and equilibrium datum. The results are presented in **Table 3**. In the aspect of classification accuracy, the consistency between the prediction results and the actual results is confirmed by the confusion matrix results, and it is proved that this model has better performance (**Figure 4A**). We verified the prediction and discrimination ability of the combined model through ROC curve analysis. The results showed that the diagnostic accuracy of stage III type 2 diabetic nephropathy and normal people was as high as 0.98 (all AUC >0.90). In the 10-fold cross-validation of the main cohort, we found that the AUC of the LR model was 0.98, tending to the upper left corner and away from the diagonal (**Figure 5A**). The combined model has the advantages of high diagnostic accuracy (92.8%) and specificity (96.5%). **Table 3** shows the performance of each model in the main queue and test queue.

**Table 3:** Performance of each model in primary cohort and test cohort.

Model name	Primary cohort					
	AUC	ACC (%)	SEN (%)	SPE (%)	PPV (%)	NPV (%)
United model	0.98	92.8	88.9	96.5	96.0	90.3
T1WI model	0.92	85.9	85.1	86.7	85.2	86.7
FS-T2 model	0.95	91.2	92.9	89.6	89.6	92.9
ADC model	0.91	80.3	74.1	86.2	83.3	78.1
T2-COR model	0.85	78.9	75.8	82.1	81.5	76.7
DWI model	0.84	75.0	74.1	75.9	74.1	75.9
	Test cohort					
United model	0.98	89.3	92.3	86.7	85.7	92.9

The united model is T1WI + T2WI + ADC model; Area under curve (AUC), Accuracy (ACC), Sensitivity (SEN), Specificity (SPE), Positive predictive value (PPV), Negative predictive value (NPV).

#### 3.2.1 Performance Assessment of Prediction Mold in Test Set

In addition to the above main data sets, we do not need preprocessing or class distribution equalization to test the performance of the joint model. In the test set, the machine learning algorithm uses the same machine learning algorithm as the primary set. In the aspect of the classifier performance in the test set, confusion matrix and ROC curve can show that the model has high classification accuracy, AUC value is 0.98 (**Figures 4B**, **5B**). ROC curve also showed that the model had at least 92% high sensitivity.

## 4 Discussion

Many studies have shown that functional magnetic resonance imaging (fMRI), namely, DWI, diffusion tensor imaging (DTI) and blood oxygen level-dependent MRI (BOLD), are safe, non-invasive imaging modalities capable of providing quantitative parameters to assess the change of renal microstructure and function. There are a limited number of studies concerning the use of renal quantitative BOLD imaging and DTI in early DN in humans (17–20). Hence, our aim in this study was to assess the capability of several sequences imaging texture features to detect early DN and build a diagnostic model. Some studies have indicated the potential use of ADC as an indicator of renal function, presenting as a lower ADC in renal insufficiency (21–28). However, there are few studies on the application of renal quantitative DWI in diabetic nephropathy (29, 30). In this study, our aim was to assess the differentiation of renal changes in patients with stage III type 2 diabetic nephropathy and normal subjects.

In this study, we extracted a total of 10 texture feature parameters from the right kidney images to construct a diagnostic model using an open source python package called PyRadiomics (2.1.0). While the figure of variables is larger than the figure of samples, we use variable selection regression. Adjusting parameter λ is an indispensability parameter in nested regression. LASSO can select the prime variable by adjusting λ. In this study, we used ten cross-validations to select λ; in order to obtain the error of mean square of each subsample, all data were randomly divided into 10 parts, of which 9 are used for model training and the other for evaluating the effectiveness of the model. In order to obtain the error of mean square of the total sample, the error of mean square of each sub-sample has plus. In order to minimize the mean square error of the entire sample, we adjust λ and select the most valuable variables. In this study, the texture features of renal damage in type 3 diabetes mellitus jerk the variation of nephritic tissue configuration and function in diabetes mellitus. MR image texture changes may be due to nephritic tubular injury and capillary reduction in diabetics, resulting in limited diffusion of hydration in renal cortex and medulla.

This study was based on non-contrast axial T1WI, FS-T2WI, and ADC sequences model to diagnose and analyze DN; DeLong test was conducted among different models to select the models for combination. In the current study, we evaluated the value of the united model based on T1WI, FS-T2WI, and ADC sequences, assessed non-invasively renal functional differences in early diabetic patients with or without microalbuminuria, and then compared with healthy control subjects. The united model had high accuracy and specificity with AUC value of 0.98. Our results demonstrated that the single sequence of FS-T2WI diagnostic model has a high diagnostic efficiency (AUC value is about 0.95), and the diagnostic accuracy, specificity and sensitivity are high, which makes a great contribution in the united model. However, T1WI and ADC sequences can provide more texture features of the kidney, which further improves the diagnostic efficiency. The reason may be that there are few layers of kidney and the texture features are limited. These MRI findings suggest that MR imaging can provide a noninvasive assessment of renal changes in the early stage of DN.

Radiological methods such as CT and MRI can be used to assess the pathological changes of abdominal organs (31). The radiomics method is expected to overcome the limitation of visual assessment of images that provide little information about histogram and texture features in an ROI (32–36). The technique has had considerable success in diagnosing and evaluating tumors (37–39). The aim of this study was to identify imaging markers and develop a model for both diagnosis of early diabetes and prediction of future diabetes by combining abdominal CT images and radiomics methods.

But, this paradigm of early DN has been further questioned because a decrease in the renal function of a patient with diabetes is not always accompanied by increased albuminuria. There is a long silent period without overt clinical signs and symptoms of nephropathy prior to the onset of microalbuminuria. Therefore, we hypothesized that the progressive pathological changes in the kidney developed after a long asymptomatic period, but this group of diabetic patients did not show proteinuria (40). Based on this assumption, the texture feature might be a new, more sensitive predictor of early DN, which could contribute to identifying these patients and intervening at an earlier time.

Though our research overcome shows the latent value of distinguishing the radiation characteristics of early renal damage and healthy kidney in diabetic patients, our research still has localizations. Firstly, this study only included the right kidney, but did not study the left kidney. We chose a kidney as a preliminary study, but in fact diabetic kidney damage often involves two kidneys, and gastrointestinal physiological movement and gas may affect the left kidney DWI measurement results. Secondly, this study is a single-center study, and the diagnostic effect of MRI-based radiomic model needs further discussion of multi-center studies. Third, there are significant statistical differences in texture feature values, but the sample content is relatively small. Fourth, only early diabetic nephropathy patients were studied; follow-up can be further studied in patients with advanced diabetic nephropathy. Because it is difficult to obtain the pathological results of early diabetic kidney damage (DKD) from clinical practice, we can compare the image texture features and pathological changes of DKD in animal experiments in the future. In summary, MRI-based radiomic features may play a promising role in the detection of early DN. As a relatively simple and noninvasive tool, it may play a role in the clinical decision-making of DN follow-up without the use of contrast agents.

## 5 Conclusions

The results of our study suggest that changes in kidneys detected by DWI may serve as indicators of early diabetic kidney disease. Early detection and intervention of diabetic renal damage have important clinical significance. We analyzed the texture features of DN III type 2 images and normal images to obtain quantitative data of texture features. Studies have found that radio tissue theory has potential application value in the diagnosis of early kidney lesion in people with diabetes. As a relatively simple, non-invasive and non-use contrast agent-free detection method, MR-based texture features have a good application prospect in the detection of early DN.

## Data Availability Statement

The original contributions presented in the study are included in the article/supplementary material. Further inquiries can be directed to the corresponding authors.

## Ethics Statement

The studies involving human participants were reviewed and approved by the China–Japan Union Hospital of Jilin University. The patients/participants provided their written informed consent to participate in this study.

## Author Contributions

YB and HC conceived the study, and FX and LF participated in its design and coordination. ZJ, SZ, ZN, and DJ helped to draft the manuscript. All authors listed have made a substantial, direct, and intellectual contribution to the work and approved it for publication.

## Funding

This work was supported by the Industrial Technology Research and Opening Up Project of Jilin Development and Reform Commission (2020C036-6).

## Conflict of Interest

Authors CH, FL, and ZS were employed by Beijing Deepwise & League of PHD Technology Co., Ltd.

The remaining authors declare that the research was conducted in the absence of any commercial or financial relationships that could be construed as a potential conflict of interest.

## Publisher’s Note

All claims expressed in this article are solely those of the authors and do not necessarily represent those of their affiliated organizations, or those of the publisher, the editors and the reviewers. Any product that may be evaluated in this article, or claim that may be made by its manufacturer, is not guaranteed or endorsed by the publisher.

## Abbreviations

DWI, Diffusion-weighted imaging; DN, Diabetic nephropathy; ACR, Albumin-to-creatinine ratio; eGFR, estimated glomerular filtration rate; MRI, Magnetic resonance imaging; ROI, Region of interest; LASSO, Least absolute shrinkage and selection operator; ROC, Receiver operating characteristic; AUC, Area under the curve; ADC, Apparent diffusion coefficient; DM, Diabetes mellitus; ICC, Intraclass correlation coefficient; GLCM, Ggray level co-occurrence matrix; GLRLM, Gray level run length matrix; GLSZM, Gray level size zone matrix; GLDM, Gray level dependence matrix; DCA, Decision curve analysis; SEN, Sensitivity; SPE, Specificity; ACC, Accuracy; PPV, Positive predictive value; PPV, Positive predictive value; NPV, Negative predictive value; CI, Confidence interval; BMI, Body mass index; fMRI, Functional magnetic resonance imaging; DTI, Diffusion tensor imaging; BOLD, Blood oxygen level-dependent MRI; CT, Computed tomography; DKD, Diabetic kidney damage; T1WI, T1-weighted imaging; T2WI, T2-weighted imaging; FS-T2WI, Fat saturation-T2-weighted imaging; LR, Logistic regression.
